# Pharmacological Applications of Bile Acids and Their Derivatives in the Treatment of Metabolic Syndrome

**DOI:** 10.3389/fphar.2018.01382

**Published:** 2018-12-03

**Authors:** Maja Ðanić, Bojan Stanimirov, Nebojša Pavlović, Svetlana Goločorbin-Kon, Hani Al-Salami, Karmen Stankov, Momir Mikov

**Affiliations:** ^1^Department of Pharmacology, Toxicology and Clinical Pharmacology, Faculty of Medicine, University of Novi Sad, Novi Sad, Serbia; ^2^Department of Biochemistry, Faculty of Medicine, University of Novi Sad, Novi Sad, Serbia; ^3^Department of Pharmacy, Faculty of Medicine, University of Novi Sad, Novi Sad, Serbia; ^4^Biotechnology and Drug Development Research Laboratory, School of Pharmacy and Biomedical Sciences, Biosciences Research Precinct, Curtin Health Innovation Research Institute, Curtin University, Perth, WA, Australia

**Keywords:** bile acids, metabolic syndrome, farnesoid X receptor, transmembrane G protein coupled receptor 5, gut microbiota, diabetes, lipoprotein, atherosclerosis

## Abstract

Apart from well-known functions of bile acids in digestion and solubilization of lipophilic nutrients and drugs in the small intestine, the emerging evidence from the past two decades identified the role of bile acids as signaling, endocrine molecules that regulate the glucose, lipid, and energy metabolism through complex and intertwined pathways that are largely mediated by activation of nuclear receptor farnesoid X receptor (FXR) and cell surface G protein-coupled receptor 1, TGR5 (also known as GPBAR1). Interactions of bile acids with the gut microbiota that result in the altered composition of circulating and intestinal bile acids pool, gut microbiota composition and modified signaling pathways, are further extending the complexity of biological functions of these steroid derivatives. Thus, bile acids signaling pathways have become attractive targets for the treatment of various metabolic diseases and metabolic syndrome opening the new potential avenue in their treatment. In addition, there is a significant effort to unveil some specific properties of bile acids relevant to their intrinsic potency and selectivity for particular receptors and to design novel modulators of these receptors with improved pharmacokinetic and pharmacodynamic profiles. This resulted in synthesis of few semi-synthetic bile acids derivatives such as 6α-ethyl-chenodeoxycholic acid (obeticholic acid, OCA), norursodeoxycholic acid (norUDCA), and 12-monoketocholic acid (12-MKC) that are proven to have positive effect in metabolic and hepato-biliary disorders. This review presents an overview of the current knowledge related to bile acids implications in glucose, lipid and energy metabolism, as well as a potential application of bile acids in metabolic syndrome treatment with future perspectives.

## Introduction

Metabolic syndrome is one of the major public health and clinical challenges worldwide. This complex condition constituted of insulin resistance, hyperinsulinemia, glucose intolerance, dyslipidemia, hypertension and obesity, increasing thus the incidence of cardiovascular diseases and mortality (Hanson et al., 2002). The global prevalence of metabolic syndrome largely varies depending on geographic and sociodemographic factors, as well as the diagnostic criteria used (McCracken et al., 2018). Results obtained from National Health and Nutrition Examination Survey estimated that 35% of adults in the United States, and as much as 50% of the people aged over 60, had a diagnosis of metabolic syndrome, with notably higher prevalence in women compared with men (Aguilar et al., 2015). The prevalence of type 2 diabetes mellitus is worldwide increasing health disorder and estimations indicate that is going to affect approximately 8% of the population by 2030 (Lam and LeRoith, 2012).

Both genetic and environmental factors such as high-calorie diet and low level of physical activity are evidently associated with metabolic syndrome (Kaur, 2014). The complexity of the metabolic syndrome etiology in combination with specific metabolic profile and lifestyle of each patient contribute to the lack of adequate efficacy with currently used drugs increasing thus demand for developing novel therapeutic alternatives with new mechanisms of actions.

Apart from classic functions of bile acids in digestion and solubilization of lipophilic nutrients and drugs in the small intestine (Mikov and Fawcett, 2006; Mircioiu et al., 2012), there is emerging body of evidence pointing to the role of bile acids and their derivatives as signaling, endocrine molecules that exert variety of metabolic effects through complex and intertwined pathways, thus becoming the attractive target for metabolic syndrome treatment (Taoka et al., 2016; Chávez-Talavera et al., 2017; de Boer et al., 2018; Molinaro et al., 2018; Shapiro et al., 2018).

This review will provide an overview of the current knowledge related to bile acids implications in glucose, lipid and energy metabolism as well as potential application of bile acids in metabolic syndrome treatment with recommendations for further studies.

## Bile Acid Biosynthesis and Enterohepatic Recirculation

Biosynthesis of bile acids represents predominant metabolic pathway of cholesterol catabolism in human organism. Conversion of cholesterol to bile acids is a multiple enzymatic process in which hepatocytes are the only cell type which contains complete set of enzymes necessary for conversion of cholesterol steroid nucleus, side chain removal and conjugation with either glycine (∼75%) or taurine (∼25%). This results in generation of two primary bile acids: cholic acid (3α,7α,12α-trihydroxy-5β-cholan-24-oic acid, CA) and chenodeoxycholic acid (3α,7α-dihydroxy-5β-cholan-24-oic acid, CDCA). Conversion of cholesterol to bile acids involves the processes of hydroxylation, C5-C6 double bond saturation, epimerization of C3 hydroxyl group and oxidative cleavage of three carbon unit of side chain (Gioiello et al., 2014). Abovementioned reactions take place in several subcellular compartments including endoplasmic reticulum, mitochondria, cytoplasm, and peroxisomes. Even though four different biosynthetic pathways have been described, the fact that intermediates in different pathways are substrates for the same enzymes, as well as the transport of bile acids and their precursors between subcellular compartments add complexity to understanding bile acid synthesis (Vaz and Ferdinandusse, 2017).

**FIGURE 1:**
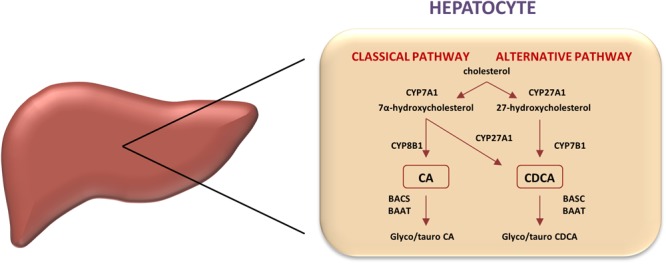
Classic and alternative pathways of bile acids synthesis. In human liver, two primary bile acids, cholic acid (CA), and chenodeoxycholic acid (CDCA), are produced. Key regulatory enzymes in both pathways are shown. Microsomal cholesterol 7α-hydroxylase (CYP7A1) catalyzes the first the rate-limiting step in the classic pathway converting cholesterol into 7-hydroxycholesterol, whereas the mitochondrial sterol 27-hydroxylase (CYP27A1) initiates the alternative pathway by converting cholesterol into 27-hydroxycholesterol, which is then 7-hydroxylated by microsomal oxysterol 7-hydroxylase (CYP7B1). CYP8B1 regulates the cholic acid synthesis in the classic pathway.

Two predominant biosynthetic pathways in humans are so called a classical (neutral) pathway and an alternative (acidic) pathway (Figure 1). Through classical pathway, 90% of primary bile acids are synthesized. Cholesterol 7α-hydroxylase (CYP7A1) is a rate-limiting enzyme, which determines quantity of bile acid pool size, catalyzing hydroxylation of cholesterol into 7α-cholesterol. Modifications of steroid nucleus and side chain render CDCA formation. In addition, hydroxylation at C12 position by sterol 12α-hydroxylase (CYP8B1) results in CA synthesis, therefore CYP8B1 is considered as an enzyme that determines hydrophilicity of bile acid pool. In classical pathway CA and CDCA are synthesized in almost equal amounts. In alternative pathway, C27 bile acids and oxysterols produced in different cell types are transported to the liver and further metabolized. Main enzymes that manage side chain shortening and 7α-hydroxylation are mitochondrial sterol 27-hydroxylase (CYP27A1) and microsomal oxysterol 7α-hydroxylase (CYP7B1) with CDCA being the major product (Li et al., 2013; Chiang, 2015). Primary bile acids are not released into biliary tree as free carboxylic acids; instead, these molecules are activated with acetyl-coenzyme A (CoA) by bile acid CoA synthetase (BACS) and formed thioester is then linked by amide bond with amino acids, either glycine or taurine (the reaction is catalyzed by bile acid-CoA: amino acid *N*-acyltransferase, BAAT). This process of conjugation resulting in bile salt formation reduces cytotoxic and membranolytic potential compared to free bile acids (Chiang, 2009). In addition, whereas unconjugated bile acids are able to diffuse across membranes, bile salts are actively transported.

Secretion of bile salts from the hepatocyte to the lumen of biliary canaliculi is mediated by two ATP-binding cassette (ABC) proteins – bile salt export pump (BSEP, *ABCB11*) and multidrug resistance-associated protein-2 (MRP2, *ABCC2*), which represent the main force for bile secretion (Dawson et al., 2009). Mutations in *ABCB11* and *ABCC2* gene result in the development of type-two progressive familial intrahepatic cholestasis, and Dubin-Johnson’s syndrome, respectively (Baghdasaryan et al., 2014). Following the meal, bile acids are released into intestinal lumen by the activity of cholecystokinin, and by forming mixed micelles, they facilitate digestion and absorption of lipophilic xenobiotics. Within intestinal lumen, microflora exerts profound effects on bile acids, with the primary aim to reduce bactericidal activity. In the lumen of ileum and colon, non-absorbed bile acids undergo deconjugation and 7α-dehydroxylation by the activity of the enzymes of anaerobic microflora – the process which results in the conversion of primary to the secondary bile acids which affect the host metabolism: deoxycholic acid (3α,12α-dihydroxy-5β-cholan-24-oic acid, DCA) and lithocholic acid (3α-hydroxy-5β-cholan-24-oic acid, LCA) as well as hydrophilic ursodeoxycholic acid (3α,7β-dihydroxy-5β-cholan-24-oic acid, UDCA) which is produced by *Clostridia* species through 7β-epimerization of CDCA) (Ramirez-Perez et al., 2017).

Deconjugated bile acids are reabsorbed passively whereas approximately 95% of ileal bile acids are efficiently reabsorbed in the distal segments of ileum by the enterocyte apical sodium-dependent bile acid transporter (ASBT, *SLC10A*2). *Trans*-enterocyte shuttle of bile acid is facilitated by the gastrotropin, intestinal bile acid-binding protein (IBAP, *FABP6*), whereas basolateral heterodimeric organic solute transporter (OST) α/β facilitates efflux into the portal circulation. Hepatocytes uptake bile salts through sodium-dependent bile acid transporter (NTCP, *SLC10A1*) whereas non-conjugated bile acids are reuptaken by hepatocytes through sodium-independent organic anion transporters (OATPs) localized in sinusoidal membrane of hepatocyte (Zwicker and Agellon, 2013). Composition of the bile acid pool, including both the conjugated bile acids/salts following release from gallbladder and deconjugated bile acids metabolized by intestinal microflora, is variable during the day, depending upon dietary habits and composition of intestinal microbiota (Ajouz et al., 2014). Significant cytotoxic propensity of LCA results in fecal excretion of this bile acid species, and small amount of hydrophobic LCA recirculated in the liver undergoes sulfoconjugation in hepatocytes by sulfotransferase 2A1 (SULT2A1) and fecal excretion (Hofmann, 2004).

**FIGURE 2:**
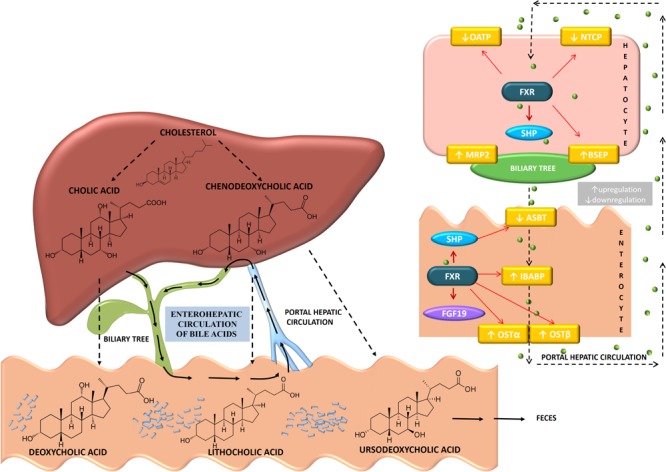
Biosynthesis and enterohepatic circulation of bile acids showing the individual transport proteins expressed in hepatocytes and enterocytes. Primary bile acids synthesized in the liver from cholesterol are cholic acid (CA) and chenodeoxycholic acid (CDCA). The primary bile acids are then conjugated with glycine or taurine in the liver and released into the gallbladder. Gut microbiota present in the intestine, by deconjugation, dehydroxylation, dehydrogenation and epimerization, modify the primary bile acids converting them to the secondary bile acids-deoxycholic acid (DCA), lithocholic acid (LCA), and ursodeoxycholic acid (UDCA). Around 95% of bile acids are reabsorbed into the distal ileum. Transporters involved in enterohepatic circulation of bile acids are apical sodium-dependent bile transporter (ASBT) present in the brush border membrane of the enterocyte, ileal bile acid-binding protein (IBABP) involved in *trans*-enterocyte shuttle of bile acid, basolateral organic solute transporters, OSTα/OSTβ that facilitate efflux into the portal circulation. Bile acids are actively transported into hepatocytes by sodium sodium-taurocholate cotransporting polypeptide (NTCP) and organic anion transporters (OATP) while bile salt export pump (BSEP) and multidrug resistance-associated protein-2 (MRP2) are involved in the process of exporting bile acids out of hepatocytes. Bile acids activation of FXR signaling pathways regulates the expression of these transporters. In the hepatocyte, bile acid activation of FXR increases SHP expression, decreases the expression of NTCP and OATP, while increases the expression of MRP2 and BSEP. In ileal enterocytes, bile acids signaling leads to upregulation of IBABP and OSTα/OSTβ and downregulation of ASBT. (Bile acid direction is presented by black dashed narrow.)

Bile acid pool is 4–14 times recycled during the day and the fraction lost through feces is compensated by *de novo* synthesis from cholesterol. Therefore, interruption of enterohepatic recirculation and increased excretion of bile acids represents effective therapeutic strategy in the treatment of hypercholesterolemia (Chiang, 2013). Biosynthesis of bile acids and their enterohepatic circulation with specific transmembrane transport proteins in enterocytes and hepatocytes and are clearly shown and explained in Figure 2.

## Bile Acids as Signaling Molecules

Enterohepatic recirculation of bile acids amplifies *trans*-hepatic flux of bile acids that activate farnesoid X receptor (FXR), the most significant transcription factor involved in the regulation of bile acid biosynthesis and transport. Additionally, *trans*-intestinal flux of reabsorbed bile acids activates intestinal FXR, regulating enterohepatic recirculation of these amphiphilic molecules. These hepatic and intestinal events prevent hepatocytes overload and accumulation of toxic concentrations of bile acids and prevent hepatocellular injury, by decreasing their own synthesis and uptake, maintaining at the same time sufficient amounts of bile acids in biliary tree and intestinal lumen for emulsification of dietary lipids. Moreover, hepatocellular uptake of bile acids is incomplete and micromolar range of concentrations spills from portal into systemic circulation through hepatic veno-venous anastomoses. These systemic concentrations are sufficient to interact with several currently identified nuclear receptors; FXR, pregnane X receptor (PXR), vitamin D receptor (VDR), and constitutive androstane receptor (CAR), as well as bile acid-coupled membrane receptor Takeda G-protein receptor-5 (TGR5, GPBAR) exerting systemic signaling effects beyond enterohepatic tissues (Gioiello et al., 2014; Comeglio et al., 2017).

Farnesoid X receptor is a metabolic nuclear receptor which primary role is to sense concentration of bile acids and prevent their accumulation to toxic concentration in hepatocyte. Human gene *FXRα* (*NR1H1*) which is located at chromosomal locus 12q23.1 encodes for FXR isoforms as a result of difference in transcription initiation site (either exon one or exon three) and alternative splicing between exons five and six (Kemper, 2011). FXR protein has been shown to be widely distributed in the organism and metabolic significance of FXR has been revealed in different cell- and tissue-types particularly by using tissue specific FXR loss-of-function rodent models (Gardmo et al., 2011). Like the other nuclear receptors, FXR contains ligand-binding domain, DNA-binding domain that binds to specific DNA sequences known as the FXR-response elements (FXREs) and co-activator and co-repressor interacting activating domains -1 and -2 (AF1, AF2) bridged by hinge region (Gioiello et al., 2014). FXR regulates the expression of target genes mostly by hetero-dimerization with retinoic acid receptor (RXR), inducing chromatin de-compaction through activating acetylation of histones or by other (epi)genetic mechanisms, resulting in the transactivation of target genes (Pavlovic et al., 2017). FXR also induces gene repression mainly through the small heterodimer partner (SHP), so called an “orphan” nuclear receptor that lacks DNA-binding domain, and binds to regulatory sequences of transcription factors leading to the transcriptional repression of target genes. In the hepatocytes, FXR-SHP axis suppresses bile acid synthesis and uptake transporters, whereas in enterocytes, FXR-SHP axis suppresses uptake and induces portal efflux of already absorbed bile acids (Ferrebee and Dawson, 2015). Ligand-dependent *trans*-activation of targeted genes by bile acids is induced by the binding of bile acid as the most potent endogenous ligands, with the CDCA being the most potent agonist, whereas hydrophilic UDCA does not activate FXR (Kemper, 2011). The potency of natural bile acids for FXR is summarized in Table 1 (according to Gioiello et al., 2014). In the intestine, FXR activates transcription of enterokine, fibroblast growth factor-19 (FGF-19 or rodent *FGF-15* ortholog) through SHP, governing post-prandial bile acid and nutrient metabolism (Mertens et al., 2017).

**Table 1:** Potency of natural bile acids for FXR biding (modified according to Gioiello et al., 2014).

Bile acid species	Free carboxylic acid	Glycine conjugated bile salt	Taurine conjugated bile salt
CDCA (primary)	4.5 μM	10 μM	10 μM
CA (primary)	>100 μM	>100 μM	>100 μM
LCA (secondary)	3.8 μM	4.7 μM	3.8 μM
DCA (secondary)	100 μM	>100 μM	>100 μM
UDCA (secondary)	>100 μM	>100 μM	>100 μM

Additionally, as endobiotics, bile acids may activate PXR (*NR1I2*, chromosome 3q13.33), which possesses the large ligand-binding pocket allowing it to interact with a wide range of structurally diverse hydrophobic compounds including drugs, dietary supplements, environmental pollutants. Non-activated PXR most commonly creates inhibitory complexes with silencing mediator for retinoic acid receptor and thyroid hormone receptor (SMRT), SHP and histone deacetylases (Pavlovic et al., 2017). Upon ligand binding, receptor undergoes conformational changes and the release of corepressor factors induces histone acetylation or recruitment of co-activating proteins like steroid receptor coactivator-1 (SRC-1) (Pavek, 2016; Buchman et al., 2018). The main bile acid ligand of PXR is LCA, its oxidized metabolite 3-keto-LCA and acetylated DCA and CA derivatives, while conjugated bile acids do not activate PXR (Carazo et al., 2017). Studies have demonstrated that PXR can alleviate the harmful effects of toxic hydrophobic bile acids such as LCA through activation of cytochromes that hydroxylate bile acids to less toxic, more hydrophilic bile acid species and by induction of conjugation enzymes, sulfotransferase SULT2A1 and uridine 5′-diphospho-glucuronosyltransferases, involved in phase two of metabolism and detoxification of bile acids (Wang et al., 2012). PXR is mostly expressed in the intestine and liver, and the main targets of this nuclear receptor are drug-metabolizing enzymes and transporter proteins. Hence, the influence of bile acids on the PXR activity may exert profound effects on the pharmacokinetics of numerous drugs and therapeutic outcome.

CAR (*NR1I3*, chromosome 11p15.5) is mainly expressed in the liver and functions predominantly as a xenobiotic sensor and transcriptional regulator of genes influencing drug disposition. Additionally, CAR is unusual among the other nuclear receptors in being constitutively active even in the absence of ligands. Besides, CAR gets its name from two androstane metabolites, which are identified as inverse agonists that repress the constitutive activity of CAR. CAR has been implicated in regulating the expression of multiple genes involved mainly in drug metabolism and disposition (Carazo et al., 2017; Pavlovic et al., 2017). CAR is a receptor for structurally diverse molecules including bile acids as endogenous ligands, with primary bile acid, CA, and keto derivatives 6-keto- and 7-keto-LCA being recognized to exert *trans*-repressive effect on CAR activity (Stedman et al., 2004; Fiorucci et al., 2010). The accumulation of bile acids during cholestasis and subsequent activation of CAR reduces hepatocellular injury by the reduction of bile acid synthesis as well as by inducing metabolizing enzymes and transporter proteins, which mediates removal of hydrophobic bile acids from hepatocytes (Li and Chiang, 2013; Gabbia et al., 2017). Both CAR and PXR cooperatively diminish bile acids toxicity. Combined loss of CAR and PXR leads to increased sensitivity to bile acid toxicity, since LCA was tolerated by wild-type and PXR-knockout mice, whereas in PXR/CAR double knockout mice, significant accumulation of serum bile acids and liver damage occurred (Uppal et al., 2005). On the other hand, the repression of CAR decreased the bile acid accumulation in cholestasis model of bile duct ligation in mice, indicating the potential of selective CAR antagonists as therapeutic strategy for cholestatic liver disease patients in the future. Even though CAR and PXR display some overlapping functions, and the loss of function of these receptors sensitizes the liver vulnerability to bile acid-mediated toxicity, these receptors do not have capacity to compensate the loss of each other (Stedman et al., 2005).

Bile acids also activate membrane G-protein-coupled receptor TGR5 through which these molecules exert non-genomic effects. TGR5 was first identified as a G-protein-coupled bile acid receptor (GPBAR1, MBAR, GPCR19) in 2002 (Maruyama et al., 2002). Upon bile acids binding, the activation of TGR5 is associated with an intracellular accumulation of cyclic AMP, adenylate cyclase activation and calcium mobilization, followed by downstream modulation of their target signaling pathways. TGR5 is ubiquitously expressed in organism, being even identified in the spinal cord and astrocytes, indicating that bile acids exert numerous previously unanticipated functions. In accordance with wide tissue distribution, bile acid-activated TGR5 receptor is involved in regulation of diverse processes in the organism, ranging from macrophage activation to glucose metabolism regulation. Being expressed in immune cells, bile acids *via* this receptor reduce production of inflammatory cytokines, whereas in the skeletal muscle and brown adipose tissue bile acids increase energy expenditure through the activity of iodothyronine deiodinase D2 – an enzyme which converts thyroxine to more potent form triiodothyronine, thus preventing obesity (Deutschmann et al., 2018). By activating TGR5 in intestinal entero-endocrine L cells bile acids stimulate the release of insulinotropic glucagon-like peptide-1 (GLP-1), regulating postprandial insulin release and blood glucose concentration (Fiorucci et al., 2009). Studies have also shown that activation of TGR5 on pancreatic β-cells stimulates glucose-induced insulin release through cyclic AMP- and calcium-dependent manner, evading stimulation of the glucagon release from α-cells (Kumar et al., 2016).

Therefore, bile acids are becoming increasingly considered as hormones which may affect various aspects of integral metabolism. However, given that bile acids activate different receptors, significant effort has been made to synthetize, characterize and novel bile acid derivatives (as well as non-bile acid synthetic ligands) with high specificity toward certain receptor, ensuing dissection of signaling pathways and uncovering novel therapeutic targets. Out of numerous bile acid derivatives, only obeticholic acid (OCA, 6-ethyl-chenodeoxycholic acid, 6-ECDCA INT-747) has currently been clinically useful and approved as a potent and specific FXR agonist with 100-fold higher affinity compared to the CDCA – endogenous FXR agonist with the highest potency (Pellicciari et al., 2002). In addition, 6α-ethyl-23(S)-methylcholic acid (INT-777) has been identified as a specific TGR5 agonist with promising effects in treatment of glucose homeostasis disorders, vascular inflammation and atherosclerosis, neuro-inflammation and hepato-gastro-intestinal and metabolic disorders (Pellicciari et al., 2009; Guo et al., 2016).

UDCA is hydrophilic bile acid which β conformation of C7 hydroxyl group interrupts hydrophobic integrity of steroid core’s hydrophobic surface, increasing hydrophilicity but significantly affecting interaction with ligand-binding pocket (LBP) of FXR, making impossible formation of hydrogen bond with tyrosine-366 of LBP and activation of FXR (Katona et al., 2007). Even though UDCA is not potent ligand for other bile acid receptors, this bile acid species exerts various therapeutic effects by different mechanism including antioxidative and anti-apoptotic functions, the role of molecular chaperone regulating endoplasmic reticulum homeostasis, protein folding and post-translational modification of proteins (Perez and Briz, 2009; Cybulsky, 2017; Mueller and Castro, 2018). UDCA modulates apoptosis by tumor suppressor p53, through complexation of p53 with an oncoprotein Mdm 2 (mouse double minute 2), preventing therefore p53 nuclear transportation and activation (Amaral et al., 2010). In addition to regulation of endoplasmic reticulum homeostasis, UDCA stabilizes mitochondrial membrane reducing pro-apoptotic potential of cell, which has profound implications in pathologies with excessive apoptosis (Chun and Low, 2012). This is particularly relevant for tauro-UDCA which has proven beneficial therapeutic effect in central nervous system in models of Parkinson’s disease, neuroinflammation, retinal degenerative diseases, spinal cord injury (Lobysheva et al., 2018; Miao et al., 2018; Rosa et al., 2018). In addition to being a “neuroprotective conjugate,” tauro-UDCA has therapeutic potential, endothelial dysfunction, protecting bile acid composition during inflammatory conditions, reducing the progression of high-fat diet-induced non-alcoholic fatty liver disease (NAFLD), ameliorating intestinal inflammation, improving function of intestinal barrier, and modulating the composition of intestinal microbiota (Walsh et al., 2016; Hou et al., 2017; Wang et al., 2018).

24-Norursodeoxycholic (norUDCA) is specific non-amidated side chain-shortened C23 homolog of UDCA. NorUDCA derivative has a property of targeting biliary canalicular lumen, being shuttled through hepatocyte and excreted in biliary tree as an anion, protonated at biliary pH. NorUDCA induces bicarbonate-rich choleresis with cholangio-protective effects. In addition, norUDCA has direct anti-inflammatory, anti-lipotoxic, anti-fibrotic and anti-proliferative properties having effects on different liver cell populations, representing one of the most promising novel therapeutic approaches targeting both the liver and the system of biliary tree at multi-cell-type and multi-factorial levels (Trauner et al., 2015). NorUDCA protects cholangiocytes from luminal injury as hydrophilic bile acid. This bile acid derivative does not undergo enterohepatic recirculation like conjugated bile salts. Namely, protonated norUDCA is a non-polar molecule that can passively diffuse through cholangiocytes into the circulation of peribiliary plexus to be re-uptaken by the liver cells and re-excreted into the bile in a process known as ‘cholehepatic shunting.’ Since for each norUDCA molecule that passes through cholangiocyte a bicarbonate anion remains in the bile, this process increases biliary bicarbonate concentration, which is known to have protective propensity in cholangiopathies as ‘bicarbonate umbrella,’ favorably changing composition of bile (Halilbasic et al., 2015). Indeed, norUDCA ameliorated both the liver and ductal injury in *Mdr2* knockout mice (Fickert et al., 2006). Opposite to UDCA, which increases intra-biliary pressure in obstructive cholestasis due to induced choleresis, norUDCA has been demonstrated not to aggravate hepatocyte’s injury in a rodent model with obstructive cholestasis, which may be explained by the induction of alternative transporter proteins (Fickert et al., 2017).

Overall, the role of bile acids has evolved to the regulatory function which mode of action includes not only receptor-mediated, but also receptor-independent cellular signaling cascades and regulation of epigenetic machinery, making these initially described intestinal emulsifiers as the novel promising therapeutic agents in a plethora of metabolic, inflammatory and even malignant diseases. The discovery that bile acids activate cell receptors, having hence intrinsic property of signaling function rather than exclusively emulsifying function, initiated the renaissance in research in the field of bile acid pharmacology and novel discoveries have been directed toward identification of receptor specific analogs. The mode of action of bile acids is complex and could be determined by several premises: namely, it is structure-dependent since small modifications in the structure significantly alter physiological/pharmacological activity; the activity of these molecules is tissue- and cell-type dependent and displays variable effects in normal and malignant tissues, depending on the context. Deciphering complex network underlying currently described effects of bile acids and their derivatives following administration in various preclinical and clinical settings, as well as the effects of administration of synthetic non-steroid bile-acid receptor modulators is beyond the scope of this article. Therefore, this article is going to provide up to date information regarding the role of bile acids and bile acid derivatives in the treatment of the components of metabolic syndrome.

## Structural and Physico-Chemical Properties of Bile Acids Related to Pharmacological Activity

Bile acids are characterized by specific chemical structure derived from a hydrophobic hydrocarbon perhydrocyclopentanophenanthrene with flexible acidic side chain and polar hydroxyl groups. Steroid nucleus with angular methyl groups at position C-18 and C-19 represents a convex hydrophobic β-face, while hydrophilic concave α-face, contains polar hydroxyl groups varying in their number and position (Monte et al., 2009). Therefore, bile acids are amphiphilic molecules that exhibit a great surface activity and tend to self-associate in aqueous solutions forming micelles as long as their concentrations are above a certain concentration, commonly called the critical micellar concentration (CMC) (Hofmann and Hagey, 2008). The key factor that determines the self-association of bile acids is their hydrophobicity that depends on chemical structure, particularly the number and orientation of hydroxyl groups. Bile acids with fewer hydroxyl groups are usually more hydrophobic and *vice versa* (Ashby et al., 2018). The most hydrophobic bile acid, based on chromatographic behavior is LCA, followed by DCA, CDCA, CA and UDCA (Jia et al., 2017). CMC values of selected bile acids reported by Roda et al. (1989) are given in Table 2. In addition to the concentration, bile acid hydrophobicity is the most important determinant of their toxicity that is associated with cell membrane damage, promotion of the generation of ROS, and eventually necrosis and apoptosis. The more hydrophobic bile acids exhibit more cytotoxic properties compared to hydrophilic bile acids (Perez and Briz, 2009). Since the bile acids have been continuously investigated for therapeutic applications, it is of high importance to take in consideration inter-species variation in bile acid metabolism and composition between animals and humans that might contribute to the discordant toxicity.

**Table 2:** Critical micellar concentrations (CMCs) of selected bile acids and their conjugates (reported by Roda et al., 1989).

Trivial name	Symbol	Substituents	CMC (0.15 M Na^+^) (mM)
**Dihydroxy bile acids**			
Chenodeoxycholic acid	CDCA	3α,7α	4
Taurochenodeoxycholic acid	TCDCA		6
Glycoursodeoxycholic acid	GCDCA		10
Ursodeoxycholic acid	UDCA	3α,7β	7
Tauroursodeoxycholic acid	TUDCA		2.2
Glycoursodeoxycholic acid	GUDCA		4
Deoxycholic acid	DCA	3α,12α	3
Taurodeoxycholic acid	TDCA		2.4
Glycodeoxycholic acid	GDCA		2
**Trihydroxy bile acids**				
Cholic acid	CA	3α,7α,12α	11
Taurocholic acid	TCA		6
Glycocholic acid	FCA		10
Ursocholic acid	UCA	3α,7β,12α	39
Tauroursocholic acid	TUCA		40
;Glycoursocholic acid	GUCA		30

As weak acids, bile acids exist in their ionized form, i.e., as bile salts, at physiological conditions. Conjugation of these compounds with either glycine or taurine improves their physico-chemical characteristics by decreasing hydrophobic characteristics and increasing the water-solubility (Hofmann and Hagey, 2008). In the intestinal tract, bile acids are mainly in the form of mixed micelles composed mostly of bile salts, lecithin, and cholesterol, reducing thus bile acid toxicity and representing significant mechanism of cholesterol transport from the liver into the intestine and its excretion from the organism (Roda et al., 1983).

Specific structure of bile acids relating to amphiphilic properties with both hydrophilic and hydrophobic regions is responsible for specific functions of these compounds (Monte et al., 2009). The most important and well-known physiological functions of bile acids are solubilization of fatty acids, cholesterol and liposoluble vitamins in the intestinal tract, thus facilitating their digestion and transport. However, there is a growing body of evidence that the list of their physiological roles is far longer and still not complete (Li and Chiang, 2015; Chávez-Talavera et al., 2017). Discovery of novel functions of bile acids as signaling molecules involved in a plethora of metabolic pathways and signaling cascades represents a driving force to design and synthesize novel selective and potent modulators of these receptors with improved pharmacokinetic and pharmacodynamic profiles. The meaningful challenge in designing agonists of these receptors is to unveil some specific properties that are relevant to their potency and selectivity for particular receptors and to distinguish the desired therapeutic effects from the undesired side effects (Pellicciari et al., 2009).

Introduction of α ethyl moiety in the C6 position of CDCA was found to dramatically increase the agonist activity and selectivity for FXR, thus resulting in discovery of OCA (Pellicciari et al., 2002; Sato et al., 2008). Furthermore, Pellicciari et al. (2009) reported that methylation at the C23-(S) position in the side chain of bile acids such as CDCA and OCA, is a key feature to improve potency and selectivity for TGR5.

Furthermore, the unique and specific structure of bile acids make them attractive compounds in the drug development process, as pharmaceutical tools and potential drug carrier systems that could improve, control and localize drug delivery (Stojančević et al., 2013). Since mid-1990s, much attention has been paid to the investigations of keto derivatives of bile acids, primarily 12-monoketocholic acid (12-MKC), as absorption enhancers through different biological membranes (Al-Salami et al., 2008, 2012; Kuhajda et al., 2009; Yang et al., 2011; Chen et al., 2012; Danic et al., 2016). Replacing hydroxyl with keto group in CA at C12 position produces less lipophilic bile acid with higher CMC and consequently with diminished cytotoxic property, but with preserved absorption-promoting activity (Chen et al., 2012).

## Traditional and Current Therapeutic Use of Bile Acids

Beneficial effects on human health and therapeutic use of bile acids have been recognized since ancient times (Mikov et al., 2006). Bear bile has been used in Traditional Chinese Medicine clinical practice for thousands of years. Bear bile was used for detoxification, reduction of inflammation, swelling, fever and pain, several liver diseases, including fibrosis, biliary cirrhosis, and even liver cancer. Bile acids were recognized as the main compounds in bear bile responsible for pharmacodynamic activity (Li et al., 2016).

Clinical studies have confirmed the beneficial effects of bile acids, particularly UDCA in a broad spectrum of cholestatic liver diseases, including primary biliary cirrhosis, pediatric cholestatic disorders, primary sclerosing cholangitis (PSC), and drug-induced cholestasis (Paumgartner and Beuers, 2002). Western medicine recognized therapeutic potential of bile acids during the 20th century. Only UDCA and CDCA were used for the treatment of cholelithiasis for several decades, however, the discovery at the end of 20th century that bile acids activate FXR brought a renaissance in the research of bile acids. The search for selective agonist of bile acid receptor resulted in the synthesis and pre-clinical studies of numerous bile acid derivatives. In the year of 2016, US Food and Drug Administration (FDA) approved the use of CA in inborn disorders of bile acid synthesis and as additional remedy in peroxisomal disorders including Zellweger cerebro-hepato-renal syndrome, as well as a local injection use of DCA for submental fat tissue contouring (Mullard, 2016). During the same year, FDA gave approval for the use of semi-synthetic potent and selective FXR agonist, OCA, for primary biliary cirrhosis therapy in patients with unsatisfactory response to UDCA administration as a monotherapy (Hirschfield et al., 2015). This is the first approval of medicine for primary biliary cirrhosis indication after more than two decades (Gerken and Nitschmann, 2017). In addition, FXR agonist OCA has been extensively investigated in clinical settings. Treatment with OCA has been shown to improve biochemical and histological parameters in patients with NAFLD (Neuschwander-Tetri et al., 2015). During phase II study in a group of patients with NAFLD and type 2 diabetes, OCA improved central and peripheral insulin sensitivity and reduced parameters of hepatic inflammation and fibrosis yielding promising results in larger studies (Mudaliar et al., 2013). NorUDCA, as a novel bile acid derivative, has been successfully implemented during phase II clinical trial in patients with PSC. The safety profile of norUDCA was similar to placebo, whereas serum parameters of cholangiocyte injury were reduced, which gives promising expectations (Fickert et al., 2017).

Many bile acids derivatives have been synthesized and characterized during past decade, and this is continuously growing and promising field of biomedical research worldwide. Today therapeutic role of bile acids appears to expand rapidly attracting the great attention in the field of metabolic syndrome treatment (Taoka et al., 2016).

## Bile Acids in Regulation of Glucose Metabolism

Over the past decade, a growing body of evidence has strongly indicated that bile acids regulate glycemic postprandial metabolism displaying also anti-diabetic signaling capacities by acting through bile acid-activating receptors, as well as by improving protein folding and function, in addition to antiapoptotic effects (Hylemon et al., 2009).

There are, however, some unsolved discrepancies in literature between obtained results related to implication of bile acids in glucose metabolism. Initial studies identifying the implication of FXR in glucose metabolism showed that bile acids or synthetic FXR-specific agonists induced the expression of the rate controlling enzyme of gluconeogenesis, phosphoenolpyruvate carboxykinase (PEPCK), increasing the total glucose output in human and rat hepatocyte, as well as in mice *in vivo* (Stayrook et al., 2005). On the contrary, Yamagata et al. (2004) have shown that bile acids suppress the expression of gluconeogenesis genes PEPCK, glucose-6-phosphatase (G6Pase) and fructose 1,6-bis phosphatase (FBP1) *via* the interaction between SHP with hepatocyte nuclear factor 4 (HNF-4) or forehead transcription factor Foxo1, in both *in vivo* and *in vitro* conditions. Decrease of PEPCK expression appears to be an attractive possibility for type 2 diabetes treatment, given that type 2 diabetes is characterized by an increased hepatic glucose output and hyperglycemia (Cariou et al., 2005). In addition, the activation of hepatic FXR leads to increased glycogen synthase activity by phosphorylation and inactivation of glycogen synthase kinase 3 (GSK3b) (Mencarelli and Fiorucci, 2010).

Zhang et al. (2006) showed that FXR deficiency in mice is associated with glucose intolerance and insulin resistance manifested by hyperglycemia, impaired glucose tolerance, and severely impaired insulin signaling in liver, muscle and adipose tissue. Accordingly, activation of FXR by the synthetic non-steroidal agonist GW4064 in mice (30 mg/kg twice a day) significantly decreased hepatic glucose production, lowered blood glucose levels, increased glycogenesis, improved insulin synthesis and secretion, and improved central (hepatic) and peripheral insulin sensitivity in animals (Zhang et al., 2006).

FXR-induced activation of insulin transcription and secretion in pancreatic β cells are regulated by different mechanisms involving both, genomic as well as non-genomic effects. Genomic effects of FXR activation are based on the induction of the glucose-dependent transcription factor krueppel-like factor 11 (KLF11), which is proven to be an essential factor for insulin gene transcription. Non-genomic effects of FXR activation in βTC6 cells relay on the increase of Akt phosphorylation and translocation of the glucose transporter type 2 (GLUT2), a member of membrane proteins that facilitates transport of glucose along a gradient of concentration at plasma membrane, increasing the glucose uptake by pancreatic β cells (Renga et al., 2010). Furthermore, FXR activation induces the expression of GLUT4 glucose transporter in the liver, which expression is found to be reduced, both in type 1 and 2 diabetic subjects (Garvey et al., 1991, 1992). Activation of FXR was able to upregulate GLUT4 expression through FXRE in the *GLUT4* gene promoter. Shen et al. (2008) reported that activation of FXR by CDCA in concentration 10 μM could induce GLUT4 transcription in 3T3-L1 and HepG2 cell lines and increase the GLUT 4 protein expression in C57BL/6J mice that were treated with CDCA (20 mg/kg/day). Overall, these changes resulted in decrease in plasma glucose level, decreased hepatic gluconeogenesis, and increased hepatic glycogen synthesis (Mencarelli and Fiorucci, 2010).

**FIGURE 3:**
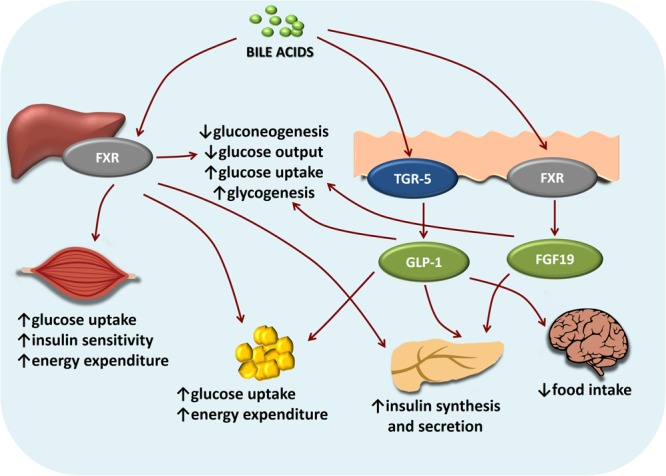
Intertwined bile acids-mediated signaling pathways in glucose metabolism. Bile acids activation of FXR and TGR-5 signaling pathways inhibits gluconeogenesis and promotes glycogen synthesis in the liver, promotes glucose-stimulated insulin release in pancreas, increases energy expenditure especially in skeletal muscles and brown adipose tissue. In the brain, bile acids-TGR5 signaling mediates satiety.

Bile acids may also affect glucose homeostasis in an FXR-independent manner (Stanimirov et al., 2012). In addition to the role in energy expenditure (discussed in further text), the activation of membrane receptor TGR5 by bile acids has been shown to increase intestinal GLP-1 secretion from entero-endocrine L cells, both *in vitro* and *in vivo*, stimulating the release of insulin from the pancreatic β-cells without the release of glucagon from α-cells, and reducing postprandial glycaemia (Katsuma et al., 2005; Kumar et al., 2012; Duboc et al., 2014). Furthermore, Maruyama et al. (2006) showed that TGR5-*null* mice have a 25% decrease in bile acid pool size, while female TGR5-*null* mice showed significant fat accumulation with body weight gain compared with that of the wild-type mice when fed a high fat diet. Figure 3 clearly shows intertwined pathways of bile acids implication in glucose metabolism mediated by FXR and TGR5 signaling.

*In vitro* and *in vivo* genetic evidence indicate a strong and causal link between the functional capacity of the endoplasmic reticulum and insulin effects. Therefore, the modulation of endoplasmic reticulum function might provide a new approach for diabetes treatment (Ozcan et al., 2006). Ozcan et al. (2006) showed that tauro-UDCA improves insulin resistance by attenuating endoplasmic reticulum stress in type 2 diabetes animals resulting in normalization of hyperglycemia, improvement of systemic insulin sensitivity and insulin action in various tissues.

The efficacy of synthetic FXR agonists as potential therapy for type 2 diabetes mellitus has been proven in an already mentioned phase II clinical trial conducted by Mudaliar et al. (2013) showing that administration of OCA to patients with both type 2 diabetes mellitus and NAFLD for 6 weeks was well-tolerated, improving insulin sensitivity, and reducing markers of liver inflammation and fibrosis.

### Bile Acid-Mediated Effects on Diabetes After Bariatric Surgical Procedures

Recently, the search for the mechanisms underlying rapid glycemic improvements following bariatric surgical procedures [Roux-en-Y gastric bypass (RYGB) and vertical sleeve gastrectomy] in patients with morbid obesity and type 2 diabetes confirmed the significant physiological effects of bile acids in glucose homeostasis (Bradley et al., 2012; Pournaras et al., 2012). These procedures caused an increase in free bile acids both in serum and in the lower intestine (Madsbad et al., 2014; Raghow, 2015). The increased concentration of free bile acids in the intestinal lumen had direct incretin effect through TGR5 – GLP-1 axis, enhancing insulin secretion, as well as by activating intestinal FXR and its direct downstream target FGF-15/19 – a postprandial hormone which improves glucose tolerance, the protein which expression is, otherwise, reduced in diabetic patients (Jansen et al., 2011; Schaap, 2012; Madsbad and Holst, 2014). Beneficial effects mediated by the bile acid receptors FXR and TGR5 following bariatric surgery were further confirmed since in the absence of FXR signaling in FXR-null mice or antagonizing GLP-1 signaling by exendin-(9-39), the beneficial effects of bariatric surgery on body weight and glucose metabolism were abolished (Salehi et al., 2011; Ryan et al., 2014). The increase in free bile acid concentrations in the lower parts of intestinal tract after RYGB creates an environment suitable for growth of bile-tolerant bacteria such as phylum *Proteobacter* taxonomic group (Osto et al., 2013). The overgrowth of *Proteobacter* results in a decrease of secondary, more toxic bile acid species, consequent increase of primary bile acid levels in plasma and pronounced incretin effect (Vrieze et al., 2014).

### Effects of Bile Acids–Gut Microbiota Interactions in Glucose Metabolism

Human intestinal tract is colonized by a diverse collection of microbes, with bacteria as the most numerous members. The composition of gut microbiota is specific to the individual but remains relatively constant over time (Stojančević et al., 2014). Alterations of the human gut microbiome can play a role in disease development (DeGruttola et al., 2016).

Intestinal microbiota regulates host metabolism by producing numerous metabolites that signal through their cognate receptors, thus affecting body weight, bile acid metabolism, pro-inflammatory activity and insulin resistance, and modulation of gut hormones (Han and Lin, 2014; Wahlstrom et al., 2016). One important class of metabolites produced by microbes are bile acids. Bacterial bile salt hydrolase (BSH), which is present in all major bacterial species in the human gut, carry out bile salt deconjugation thus increasing the resistance to bile toxicity, while 7α-dehydroxylases convert the primary bile acids CA and CDCA to the secondary bile acids DCA and LCA, respectively (Wahlstrom et al., 2016). Other bile salt conversions performed by the intestinal microbiota are oxidation and epimerization of hydroxyl groups, 7-dehydroxylation, esterification and desulfation, thus contributing to the great chemical diversity and more hydrophobic bile acid pool (Ridlon et al., 2016). Besides, bile acids can modulate gut microbiota composition both, directly, by destroying the structures of bacterial membranes, and indirectly, *via* FXR activation by promoting the transcription of antimicrobial agents such as iNOS and IL-18 in the small intestine and thereby inhibiting bacterial growth (Inagaki et al., 2006; Wahlstrom et al., 2016).

Considering the complex relationship of bile acids with intestinal microbiota, it is not surprising that in addition to the changes in the composition of bile acids, diabetic patients also have different composition and activity of intestinal microbiota comparing to healthy individuals (Quercia et al., 2014). Both type 1 and type 2 diabetes mellitus are associated with a decrease in overall microbial diversity, characterized with reduction of *Firmicutes* and butyrate-producing bacteria, as well as a disturbed intestine barrier function and increased gut permeability (DeGruttola et al., 2016; Knip and Siljander, 2016). Supplementation with probiotic bacteria resulted in beneficial modulation of host’s metabolism in terms of stimulated GLP-1 secretion by specific bacterial metabolites such as short chain fatty acids (SCFAs) through GPR41/43-dependent mechanism (Everard and Cani, 2014). Additionally, administration of probiotic bacteria reduced the food intake and protected from body weight gain and insulin resistance in animal models of obesity and diabetes (Yadav et al., 2013). Studies performed by our group have demonstrated that a multi-therapeutic approach using combination of probiotics and bile acids as adjunct therapy in a rat model of diabetes mellitus exerted even better glycemic regulation and resulted in the alleviation of complications compared to each treatment alone (Al-Salami et al., 2008, 2012). Synergistic effects of bile acids, probiotics and current antidiabetic therapy are reviewed in detail by Mikov et al. (2017) pointing to the potential application of this combination in metabolic disorders with special emphasis on diabetes mellitus.

Thus, the therapeutic manipulation of the intestinal microbiota by probiotic supplementation with secondary effects on bile acid pool composition represents the attractive and promising strategies for such conditions. Although further studies are highly recommended to unveil the exact mechanisms responsible for beneficial effects of bile acids-probiotic co-administration, activation of complex FXR and TGR5 signaling pathways is proposed as one of possible explanations.

## Bile Acids and Lipid Metabolism

As a main product of cholesterol catabolism, bile acids exert profound effects, not only on cholesterol metabolism, but also on the metabolism of triacylglycerols, regulating therefore metabolism of various lipoprotein species. Increased synthesis of bile acids increases the utilization of cholesterol as a substrate. By activating FXR bile acids inhibit CYP7A1 – the rate limiting enzyme of bile acid synthesis and cholesterol catabolism in the hepatocytes. In accordance, long term supplementation of either 750 mg or 375 mg/day of CDCA in patients with gallstone disease results in a modest increase in low density lipoprotein cholesterol (LDL) level (Schoenfield and Lachin, 1981). The increase in LDL occurred in 85.2% of patients receiving 750 mg/day, and, in 82.8% of patients receiving 375 mg/day, however, the increase of 67.0% was recorded in a group of patients receiving placebo, possibly as a consequence of the main disease. On the other hand, *in vitro* studies demonstrate that FXR agonist CDCA (250 μM) stabilizes mRNA of LDL receptor and increases LDL receptor activity in human cultured hepatic cell line increasing thus the uptake and clearance of LDL particles (Nakahara et al., 2002). Changes in circulating cholesterol level through the FXR activation *in vivo* are distinct among rodent models and humans. Namely, CYP7a1 expression in rodents, is opposite to the humans regulated by two nuclear receptors, the liver X receptor-α (LXR-α) and FXR, both of which abundantly expressed in the liver. LXR-α may be activated by cholesterol derivatives including 24(S),25-epoxycholesterol and 24(S)-hydroxycholesterol, and following its activation LXR interacts with a response element within the *CYP7a1* promoter, stimulating thereby gene expression. The translation of effects observed in rodents has been puzzling since in these species circulating cholesterol is predominantly packed in the form of HDL lipoproteins, opposing to LDL species dominant in human. In chimeric mice whose livers mostly contain human hepatocytes and a “humanized” lipoprotein profile, treatment with potent specific FXR agonist, semi-synthetic bile acid derivative OCA (10 mg/kg/day), results in the increase of circulating LDL and HDL reduction, similarly to the FXR activation in humans. The increase in LDL correlated with decreased sterol regulatory element-binding protein-2 (SREBP-2) activity and its target gene expression, including a significant downregulation in the expression of LDL receptor protein (Papazyan et al., 2018). The administration of OCA, 25 or 50 mg/day for 2 weeks, during clinical trials resulted in similar effects (Mudaliar et al., 2013; Walters et al., 2015).

The reduction in HDL level by FXR activation [using chow supplemented with 0.5% w/w taurocholic acid (TLCA) for a 6 days] may be explained by the repression of the apolipoprotein A-I gene, as well as the hetero-exchange of cholesteryl esters and triacylglycerols between plasma HDL and ApoB-containing lipoproteins by inducing cholesteryl ester-transfer protein expression (Lambert et al., 2003; Gautier et al., 2013). On the other hand, FXR targeting may also reinforce the reverse transport of cholesterol, a process in the cholesterol transport from peripheral tissues and cells to the hepatocytes and biliary system, in order to eliminate cholesterol through intestinal route. Northern blot analysis of liver specimens of C57BL/6 male mice, fed by 1% CA supplemented diet for a month, revealed that abovementioned effects are mediated *via* phospholipid-transfer protein, a protein which mediates the delivery of phospholipids and cholesterol from LDL to HDL lipoprotein particles. Observed effects are also a consequence of the changes in expression of the scavenger receptor-B1 (SR-B1), which is involved in the recognition of HDL particles and their uptake by the hepatocytes (Urizar et al., 2000). Also, the activation of direct FXR target, enterokine FGF15/19 has been shown to stimulate robust secretion of cholesterol into the intestinal lumen *via* the sterol-exporting heterodimer adenosine triphosphate (ATP)-binding cassette subfamily G member 5/8 (ABCG5/G8) in mice (de Boer et al., 2017). This finding has potential implication in developing strategies aimed at reduction of intestinal cholesterol resorption. The administration of 10 or 25 mg of OCA per day in healthy volunteers induced sustained elevation of serum LDL concentration and reduction of HDL, with a slight elevation of total cholesterol level independently of dose (Pencek et al., 2016). Similar changes were noted in patients with type 2 diabetes mellitus as well as in patients with biopsy-confirmed non-alcoholic steatohepatitis (Mudaliar et al., 2013; Neuschwander-Tetri et al., 2015). However, in patients with primary biliary cholangitis treated with 5–10 mg of OCA per day for 1 year, the rise in LDL and reduction in HDL level was smaller in amplitude and transient (Nevens et al., 2016). Effects of FXR activation by repeated administration of 5, 10, or 25 mg OCA per day for either 2 weeks or 20 days have been estimated in clinical trial recruiting healthy volunteers (Pencek et al., 2016). The results observed in this study confirmed the results of previous clinical studies in terms of slight disturbances in lipid status; namely, treatment with OCA induced a dose-independent reduction in HDL, as a result of reduction in small and medium HDL particles concentration, and increase in LDL cholesterol level (Pencek et al., 2016). Even though the activation of FXR by bile acids and bile acid derivatives may induce potentially pro-atherogenic phenotype in terms of the shift in lipoprotein fractions, large randomized multicenter clinical studies are highly needed to elucidate clinical impact of such dyslipidemic effects treated with bile acids or bile acid derivatives.

On the other hand, bile acids supplementation with 0.5% (w/w) CA for 8 weeks has been shown to modulate triacylglycerol metabolism through several distinct mechanisms, predominantly *via* FXR activation. By activating FXR bile acids repress triacylglycerol synthesis *de novo* through FXR downstream target, SHP-mediated inhibition of transcription of sterol regulatory element binding protein-1c (SREBP-1c), a key factor that controls transcription of several genes regulating fatty acids and triacylglycerol synthesis, including a master lipogenic enzyme in the liver, fatty acid synthase (Watanabe et al., 2004). FXR activation has also been demonstrated to induce the expression of nuclear receptor peroxisome-proliferator-activated receptor-α (PPAR-α) and of pyruvate dehydrogenase kinase isoenzyme-4 promoting fatty acid oxidation, whereas by SHP-mediated inhibition of the microsomal triacylglycerol transfer protein expression to reduce the VLDL assembly (Pan et al., 2010). Moreover, FXR promotes the activity of enzyme anchored to the luminal surface of vascular endothelial cells, a lipoprotein lipase (LPL) catalyzing therefore hydrolysis of triacylglycerols by inducing expression of LPL-activating apoC-II in C57BL/6J mice fed for 3 weeks with diet containing 0.5% sodium cholate. Additionally, FXR activation induced the expression of the VLDL receptor, facilitating the clearance of VLDL particles in HepG2 cells incubated with 50 μM of CDCA for 24 h (Jiao et al., 2015). In addition to reduce symptoms of bile acid diarrhea in patients, oral supplementation of 25 mg of OCA per day for 2 weeks resulted in increase in FGF-19 level and triacylglycerol decrease, which implicates the potential role of FGF-19 on triacylglycerol metabolism (Walters et al., 2015).

The additional complexity of signaling network between bile acids and triacylglycerol network arises upon interactions with intestinal microbiota, which is commonly changed in Western-type diet fed persons as well as in obese. Indeed, even short-term administration of oral non-absorbable antibiotics resulted in intestinal dysbiosis followed by the decrease in amounts of secondary bile acids DCA and LCA liver concentrations, and reduced serum triacylglycerol level, which did not recover even after bile acid supplementation (Kuno et al., 2018). The results of the study suggest that secondary bile acids produced by intestinal bacterial species exert significant regulatory role in maintaining serum triacylglycerol levels and metabolism in the host.

## The Role of Bile Acids in Non-Alcoholic Fatty Liver Disease

Non-alcoholic fatty liver disease, characterized by excess triglyceride accumulation within hepatocytes, or steatosis, is the most prevalent chronic liver disease. Simple steatosis is regarded as to as relatively benign and non-progressive while non-alcoholic steatohepatitis (NASH) is considered to be the most serious form of NAFLD, characterized by hepatocellular injury, chronic inflammation, and a higher risk of end stage liver diseases such as cirrhosis and liver cancer (Wree et al., 2013; Benedict and Zhang, 2017). NAFLD is strongly associated with the components of metabolic syndrome, mainly type 2 diabetes mellitus and obesity, being thus the important risk factor for both hepatic and cardiovascular mortality (Demir et al., 2015). NAFLD is the most prevalent chronic liver disease, which affects up to 40% of the population, and nearly 30% of patients with NAFLD progress NASH (Sanyal, 2011). In addition, both NAFLD and type 2 diabetes mellitus are dominant health disorders associated with the issue of global overweight and obesity epidemic (Feneberg and Malfertheiner, 2012).

Non-alcoholic fatty liver disease is therefore categorized as a hepatic manifestation of metabolic syndrome. NASH represents advanced grade of NAFLD characterized by hepatocellular injury, inflammatory infiltrate with deposition of collagen. Insulin resistance is known as fundamental feature in the pathogenesis of type 2 diabetes mellitus as well as in NAFLD, being also considered a principal disorder in the initiation and development of NASH (Kitade et al., 2017). In addition to insulin resistance, oxidative stress, hypoadiponectinemia, and visceral adiposity contribute to the progression to NASH (Gruben et al., 2014). The accumulation of triacylglycerols in hepatocytes is a result of lipolysis due to the insulin resistance, increased fatty acid tissue from mal-functional adipose tissue, impaired mitochondrial fatty acid oxidation or impaired VLDL particles assembly and export, as well as by activating inflammatory pathways (Bellanti et al., 2018).

The positive effects of bile acids-mediated FXR and TGR5 activation in a wide range of metabolic processes, including glucose metabolism and insulin signaling, triglyceride and cholesterol metabolism as well as the inflammation, put the focus of research interest on bile acids to develop effective therapeutic strategies for this liver-associated metabolic disease. Due to its characterization and pre-clinical evaluation, OCA has become one of the most commonly ligands used for decoding FXR signaling network both *in vivo* and *in vitro*. During preclinical studies OCA administration had significant beneficial effects in numerous disorders of enterohepatic system including improvement of estrogen-induced cholestasis, liver fibrosis, NASH, insulin signaling, portal hypertension, reduction of intestinal inflammation and improvement of ileal barrier function during cholestasis, improved bile acid-induced chronic diarrhea (Fiorucci et al., 2004, 2005; Adorini et al., 2012; Verbeke et al., 2014; Walters et al., 2015). *Per os* administration of 25 mg of OCA for 72 weeks has been demonstrated to improve histological features of the livers of patients with NASH during phase two of FLINT clinical trial (Neuschwander-Tetri et al., 2015). Beneficial effects of FXR activation in patient with NAFLD include improved central insulin sensitivity in the liver and consequently increased glycogen synthesis, decrease in *de novo* lipogenesis in liver, improved insulin sensitivity adipose tissue (decreasing lipotoxic effects) and improved function of peroxisome proliferator-activated receptor-gamma (PPAR-γ) and PPAR-α, regulating fatty acid and glucose metabolism both in adipose tissue and liver (Fiorucci et al., 2007). The diet supplementation with OCA (10 mg/kg/day) to fast food diet animal model of NASH, mimicking the metabolic syndrome features ablated micro RNA-21 (miR21) and activated PPAR-α that resulted in significant steatosis reduction, inflammation and lipo-apoptosis, unraveling restoration of miR21/PPAR-α axis in liver and muscle tissue by FXR and OCA (Rodrigues et al., 2017). The activation of FXR by OCA (10 mg/kg/day, administered by oral gavage for 7 weeks) and of TGR5 by 8-week-long diet intervention containing 30 mg/kg/day of INT-777, improved liver steatosis and insulin resistance in rodent model of NAFLD and obesity (Thomas et al., 2009; Cipriani et al., 2010). The administration of OCA (25, 50 mg/day *per os* during 6 weeks) in patients with type two diabetes mellitus and NAFLD significantly reduced body weight, improved insulin sensitivity and reduced serum level of gamma-glutamyltransferase, whereas increase in serum FGF-19 intestinal enterokine confirmed the activation of FXR in the patients (Mudaliar et al., 2013). Additionally, OCA (10 mg/kg) prevented hepatic inflammation by preventing detrimental nuclear factor κB-mediated immunomodulation and inflammation. Also, through inhibition of hepatic stellate cells activation, incubation with 10 μM of OCA prevented progression toward liver fibrosis and development of cirrhosis (Goto et al., 2018). Treatment with this FXR agonist resulted in improved biochemical and liver histological features in patients with NASH. These functions indicate that FXR is an attractive therapeutic target for liver diseases (Massafra and van Mil, 2018). Further large randomized clinical studies are highly desirable to confirm effects of “knocking on the FXR door” as a potential therapeutic approach in which change in bile acid pool size and composition may be exploited for the treatment of metabolic disorders.

## Bile Acids and Atherosclerosis

In addition to the systemic effects on serum lipid profile, bile acids may exert anti-atherosclerosis effects *via* FXR directly act at the level of the arterial wall. Vascular wall is not typically involved in bile acids metabolism, however, FXR has been found to be expressed in the endothelial cells and vascular smooth muscle cells (Mencarelli and Fiorucci, 2010). Activation of FXR by circulating bile acids may have a beneficial effect on vascular tone by suppressing the potent vasoconstrictive peptide, endothelin-1, and by inducing the production of the vasodilating agent, nitric oxide (NO). *In vitro* incubation of rat pulmonary artery endothelial cells with CDCA (6.25–50 μM) activated FXR in these cells and reduced expression of endothelin-1 in a concentration-dependent manner. Similarly, the incubation of rat pulmonary microvasculature endothelial cells and bovine aortic endothelial cells with CDCA (12.5 and 50 μM) resulted in increase in endothelial nitric oxide synthase (eNOS) level through transcriptional activation of the *eNOS* gene promoter by FXR (He et al., 2006; Li et al., 2008). In addition, FXR activation increases the production of physiological vaso-relaxing molecule, hydrogen sulfide (H_2_S) by activating cystathionase (CSE), the main enzyme in the *trans*-sulfuration pathway. CSE has been shown to be FXR target gene both *in vitro* (following incubation of HepG2 cells with 10 μM for 18 h) and *in vivo* (5 mg/kg of OCA administered intraperitoneally to C57BL/6j mice), reducing the portal pressure and attenuating endothelial dysfunction in the model of isolated and perfused cirrhotic rat livers (Renga et al., 2009).

The incubation of rat aortic smooth muscle cells in medium containing 30 μM of OCA and subsequent FXR activation has been shown to inhibit inflammatory responses as well as migration of vascular smooth muscle cells by inhibiting interleukin 1β-induced expression of inducible nitric oxide synthase (iNOS) and cyclooxygenase-2 (COX-2) genes, by reducing NF-κB activation. The same authors reported that NF-κB activation by IL-1β is downregulated by FXR and SHP. FXR ligands also were shown to reduce platelet-derived growth factor-β (PDGF-β) migration of rodent and human aortic vascular smooth muscle cells (Li et al., 2007). However, *in vitro* incubation of HUVEC cells with CDCA (100 μM), DCA (100 μM), and LCA (33 μM) induced the expression of adhesion molecules, such as intracellular adhesion molecule-1 (ICAM-1), vascular cellular adhesion molecule-1 (VCAM-1) and E-selectin, promoting adhesion of monocytes to endothelial cells – the pivotal step in initiating the process of atherosclerosis (Qin et al., 2006).

Pharmacological activation of TGR5 by dietary supplementation with 6α-ethyl-23(S)-methyl-cholic acid (INT-777 30 mg/kg/day) for 12 weeks in LDL receptor-knockout mice resulted in inhibition of vascular lesion formation by reducing macrophage inflammation and lipid uptake (Pols et al., 2011). TGR5, which is abundantly expressed in macrophages has been responsive to INT-777 treatment (30 μM) that inhibited cytokine production through cAMP-NF-κB signaling pathway (Pols et al., 2011). Therefore, macrophage inflammation – a central event in the majority of aspects contributing to development of the atherosclerosis, from the initiation of atherosclerosis up to plaque formation and rupture, which results in the initiation of the formation of thrombus, could be prevented by circulatory bile acids and bile acid derivatives as the agents that could affect atherosclerosis development on pleiotropic fashion.

Semi-synthetic bile acid derivative INT-767 (α-ethyl-24-nor-5β-cholane-3α,7α,23-triol-23 sulfate sodium salt) is a dual FXR/TGR5 agonist. Treatment of ApoE- and LDL receptor- knockout mice fed a Western-type diet supplemented with 30 mg/kg of INT-767 reduced atherosclerotic plaque formation downregulating the expression of pro-inflammatory cytokines and chemokines in aortic tissue (Miyazaki-Anzai et al., 2014). Treatment of ApoE knockout mice with INT-767 has also resulted in decreased levels of interleukins IL-1β, IL-6, IL-8, and IL-12 and reduced aortic expression of inflammatory mediators such as TNF-α, IL-6, IL-1β, and MCP-1. The observed anti-inflammatory effects of INT-767 rely on the activation of TGR5 but not of FXR (Miyazaki-Anzai et al., 2014).

In addition, the incubation of TGR5 expressed endothelium of the bovine aorta with TLCA (10–100 μM) resulted in dose-dependent nitric oxide synthesis (Kida et al., 2013). Among other bile acids, LCA (100 μM) also significantly increased nitric oxide production, however, incubation with DCA (100 μM) and CDCA (100 μM) only marginally increased NO production. These results are in agreement with the potency of different bile salts as TGR5 ligands to increase cAMP production in the following order: TLCA > LCA > DCA > CDCA. The established efficiency and good safety profile of UDCA in treatment of hepato-biliary disorders inspired research on the potential benefit of UDCA administration in treating pathological conditions affecting other systems of organs, e.g., cardiac disorders such as atherosclerosis and myocardial infarction. Six weeks long UDCA supplementation (13–19 mg/kg, administered twice a day) in patients with coronary artery disease improved endothelium-dependent nitric oxide-independent vasodilatation (Sinisalo et al., 1999). In addition, UDCA exerts *in vitro* anti-atherogenic effects in a model of diabetic atherosclerosis by inhibiting non-enzymatic glycation and oxidation of proteins and lipids in diabetes, which is confirmed by incubation of Raw 264.7 and HUVEC cells with 100 μM of UDCA (Chung et al., 2016). UDCA ameliorated endoplasmic reticulum stress and the downstream signaling pathway thereof in endothelial cells and aortic tissues of diabetic ApoE knockout mice, and inhibited reactive oxygen species production through induction Nrf2 as a main transcription factor stimulating transcription and synthesis of antioxidative enzymes, and inhibited NF-κB and Janus N-terminal kinase (JNK)-mediated vascular endothelial inflammation. Also, UDCA suppressed foam cell formation through upregulation of ABCA1 and ABCG1 expression, reduction of hyperglycemia-induced receptor for advanced glycation end-product (RAGE) expression, and suppression of macrophage inflammatory responses, significantly ameliorating atherosclerosis (Chung et al., 2016). These findings indicate that UDCA as a commonly used chemical chaperone could be beneficial therapeutic agent for prevention or treatment of diabetic atherosclerosis. Furthermore, UDCA (100 μM) expressed anti-atherogenic effects by blocking the endoplasmic reticulum stress and inflammatory response in endothelial cells induced by disturbed flow. *In vivo*, UDCA supplementation (∼400–600 mg/kg/day) abolished development of atherosclerotic lesions in a ApoE KO murine model with disturbed flow-induced atherosclerosis (by partial ligation of the left coronary artery) fed on high-fat-diet, and observed effects are at least partially mediated by inhibiting the activation of endoplasmic stress downstream signaling through XBP-1 and CHOP, and down-regulating expression of adhesion molecules (Chung et al., 2015).

On the other hand, elevated circulating levels of secondary bile acid, DCA (even at *in vitro* concentration of 5 μM), induced by high-fat diet have been shown to induce vascular smooth muscle cell proliferation and migration by upregulating JNK and platelet-derived growth factor β-receptor (Shimizu et al., 2014). Observed dysfunction of endothelial cells in Western-type diet subjects may the result of long-term change in intestinal microbiota composition and increased production of DCA by enterobacteria, which indicates that disturbances in intestinal microbiota may play role in the pathogenesis of cardiometabolic disorders by changing bile acid pool composition. At the same time this offers potential therapeutic/preventive strategy by manipulating intestinal microflora composition and therefore the pool size and composition of bile acids.

**FIGURE 4:**
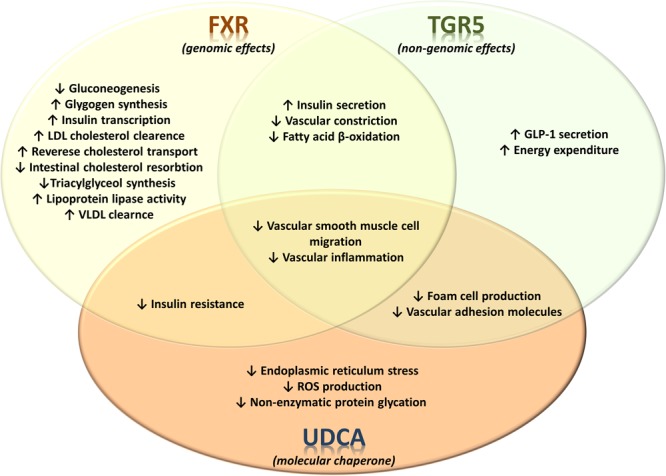
Receptor-dependent and receptor-independent regulation of metabolic and signaling pathways by bile acids in metabolic syndrome. The effects of bile acid-activated receptors FXR and TGR5 on glucose, lipid and energy metabolism as well as on vascular events associated with atherosclerosis. FXR and TGR5 have significant number of currently identified overlapping effects. On the other hand, the effects of UDCA are not associated with activation of these bile acid receptors, instead UDCA exerts various physiological/pharmacological effects which are associated with its specific structural properties.

Overall, the influence of bile acids has promising therapeutic effects in different models of atherosclerosis and dyslipidemia, the features of metabolic syndrome. The observed effects are both FXR- and TGR5- dependent and independent (summarized in Figure 4) forcing the need for developing novel bile acid derivatives as potential therapeutic agents. Given the differences between bile acid pool and composition in rodents and humans, tissue- and cell-specific activity of bile acids and context dependency on the mode of their action, the translation of obtained knowledge in preclinical settings could not be straightforwardly implemented in clinical settings. Therefore, significant effort should be carried out to identify and characterize novel bile acid derivatives as useful pharmacodynamic agents and therapeutics in the future.

## Bile Acids, Obesity, and Cardiometabolic Diseases

Obesity, a central facet of metabolic syndrome, represents a major health issue in both developed and developing countries, being associated with several comorbidities as independent risk factors for cardiovascular disease. In addition to adipose tissue excess, obese patients often display dysfunctional adipocytes. Obesity is additionally associated with dyslipidemia and elevated circulating levels of cholesterol, triacylglycerols and free fatty acids, and disturbances in lipid metabolism result in ectopic lipid deposits leading to hepatic steatosis and consequently to NAFLD. Subsequent “spill over” of free fatty acids from insulin resistant, dysfunctional adipose tissue initiates a phenomena of lipotoxicity caused by the accumulation of triacylglycerol-derived toxic metabolites in non-adipose tissues, inducing therefore inflammatory pathways, cellular dysfunction including insulin resistance, followed by impaired glucose control and type 2 diabetes (Mendez-Sanchez et al., 2018). Obesity is also associated with hemodynamic alterations and arterial hypertension which contribute to cardiac morphology changes predisposing to impairment of ventricular function and heart failure (Alpert et al., 2018).

Increased circulating bile acid level in obese individuals has been found to positively correlate with body mass index (Prinz et al., 2015). Also, insulin resistance is associated with Foxo-1-mediated downregulation of CYP8B1 resulting in depletion of 12α-hydroxylated bile acid pool, which may be explained by hyperglycemia-induced Foxo degradation, and therefore a lack to activate *CYP8B1*, whereas in type 2 diabetic patients concentration of DCA has been found to be elevated (Brufau et al., 2010; Haeusler et al., 2013). Thus, interventions manipulating bile acid pool composition could represent novel therapeutic strategies in insulin resistance. Changes in bile acid pool size and composition following bariatric surgery in obese individuals are reflected in improved metabolic homeostasis (Penney et al., 2015). It is therefore, reasonable to assume that supplementation with bile acids or bile acid derivatives, changing bile acid signaling, could be considered as potential cardioprotective intervention improving metabolism and decreasing inflammation level. Indeed, the activation of TGR5 in macrophages and endothelial cells by micromolar levels of circulating bile acids both during fasting and in postprandial state when bile circulating bile acids reach concentration peak, exert anti-atherogenic effects and inhibit atherosclerosis and coronary artery disease (Steiner et al., 2011). TGR5 receptor, expressed in adipocytes regulates energy expenditure and body weight (van Nierop et al., 2017). Bile acid-induced GLP1 also exerts beneficial effects on endothelial function, blood pressure, myocardial metabolism, left ventricular ejection fraction, atherosclerosis and response to oxidative injury induced by ischemia-reperfusion (Kang and Jung, 2016). RYGBP procedure in obese patients results in increase in GLP1 level having therefore cardioprotective effects. In addition, increased intestinal flux of bile acids following RYGBP leads to the activation of intestinal FXR and its downstream target, an enterokine FGF15/19, which has been shown to repress apo(a) gene expression by ERK1/2 phosphorylation-mediated cascade, reducing circulating level of highly atherogenic lipoprotein(a) (Chennamsetty et al., 2012). The administration of recombinant human FGF19 or FGF19 overexpression in leptin deficient diabetic mice was shown to reduce weight gain, reverse diabetes, increase lipid oxidation rate decreasing also hepatic triacylglycerol content (Fu et al., 2004). Bariatric surgery procedures also result in increase in circulating levels of bile acid species, both in fasted and postprandial state, as well as qualitative changes in bile acid pool composition, due to increased hepatic synthesis and reabsorption and reduced intestinal elimination, or by changes in microbiota composition (De Giorgi et al., 2015; Dutia et al., 2016). In accordance, the increase in concentration of bile acids both in intestinal lumen and systemic circulation, as potent ligands for FXR and TGR5, mediates the improvement of metabolic rate, glucose and lipid metabolism, increases thermogenesis resulting in reduction of body weight, ameliorates body-wide inflammation, and even promotes brown adipose tissue browning (Fang et al., 2015). These metabolic improvements implicate that change in bile acid composition or pool size by pharmacological approach or metabolic surgery affects systemic metabolism with favorable outcome, suggesting novel therapeutic approach in treating obesity and components of metabolic syndrome. However, given that bile acids activate multiple nuclear receptors and possibly more than one GPCR, careful dissection and in depth evaluation of bile acid-mediated signaling pathways on tissue specific manner should provide useful information in future development of novel specific and selective bile acid derivatives as novel therapeutic agents in treatment of metabolic syndrome.

## Conclusion

Traditional approaches, such as diet and increased physical activity, have shown to be insufficient in decreasing the prevalence of metabolic diseases that are becoming more and more common in overall population. The emerging evidence in the past decade has pointed to the role of bile acids as signaling, endocrine molecules that regulate the glucose, lipid, and energy metabolism through complex and interrelated pathways that mainly include FXR and TGR5 signaling cascade. Thus, the modulation of these signaling pathways by using bile acids and their derivatives and by co-administration with probiotic bacteria with secondary effects on bile acid pool composition has become an attractive area in modern research offering a new approach for metabolic syndrome treatment. However, many of the obtained results derived from studies carried out in animal models that should be taken into consideration during interpretation of results due to major differences in bile acid metabolism and gut microbiota composition between animals and humans. Additionally, interindividual differences in gut microbiota composition, i.e., specific bacterial *fingerprint* in certain individuals contributes to highly person-specific bile acids profiles as well, which differentially influence pathogenesis of disease, and conceivably the response to bile acid-related preventive and therapeutic interventions, requiring further studies and elucidation. From a therapeutic point of view, a more in depth insight metabolic effects of each of the natural and synthetic bile acid species *in vivo* is highly desirable. Probiotics, as potential strategy to modulate the composition of gut microbiota, should be further studied to help the restoration of bile acid metabolism and potentially aid in the treatment of metabolic disorders. Future research should be relied on metabolomic, proteomic and lipidomic approaches in both healthy and diseased populations in order to identify bile acid-related biomarkers that may be valuable for prediction of bile acid related therapy of different metabolic disorders.

To conclude, targeting the interactions between bile acids, microbiota, and bile acid receptors signaling seems to derive a promising approach for the treatment of metabolic diseases, but additional detailed pre-clinical research and translation of obtained knowledge in clinical studies are highly recommended in order to confirm the efficacy of bile acids and bile acid derivatives in such conditions.

## Author Contributions

MÐ and BS reviewed the literature and drafted the manuscript. MM and HA-S conceived the idea. All authors edited, revised, and approved the final version of this review.

## Conflict of Interest Statement

The authors declare that the research was conducted in the absence of any commercial or financial relationships that could be construed as a potential conflict of interest.
